# The impact of aging-induced gut microbiome dysbiosis on dendritic cells and lung diseases

**DOI:** 10.1080/19490976.2023.2290643

**Published:** 2023-12-12

**Authors:** Jonaid Ahmad Malik, Mohammad Adeel Zafar, Taruna Lamba, Sidhanta Nanda, Mohammad Affan Khan, Javed Naim Agrewala

**Affiliations:** Department of Biomedical Engineering, Indian Institute of Ropar, Rupnagar, Punjab, India

**Keywords:** Aging, gut microbiome, dendritic cells, lung diseases

## Abstract

Aging is an inevitable natural process that impacts every individual, and understanding its effect on the gut microbiome and dendritic cell (DC) functionality in elderly subjects is crucial. DCs are vital antigen-presenting cells (APCs) that orchestrate the immune response, maintaining immune tolerance to self-antigens and bridging innate and adaptive immunity. With aging, there is a shift toward nonspecific innate immunity, resulting in a decline in adaptive immune responses. This alteration raises significant concerns about managing the health of an elderly population. However, the precise impact of aging and microbiome changes on DC function and their implications in lung-associated diseases remain relatively understudied. To illuminate this subject, we will discuss recent advancements in understanding the connections between aging, gut dysbiosis, DCs, and lung diseases. Emphasizing the key concepts linking age-related gut microbiome changes and DC functions, we will focus on their relevance to overall health and immune response in elderly individuals. This article aims to improve our understanding of the intricate relationship between aging, gut microbiome, and DCs, potentially benefiting the management of age-associated diseases and promoting healthy aging.

## Introduction

The microbiome consists of diverse microorganisms colonizing the skin, mucosal compartments, and the gut^1^. The microbiome has been showing active involvement in the vital functions of the human body, such as immunity, circadian rhythmicity, metabolism, and nutritional responses^2^. The human immune system is a complex network present in all tissues of the human body. The immune system plays a vital role in the host’s defense against harmful exogenous and endogenous molecules for maintaining homeostasis. From an ecological perspective, the commensal microorganisms and mammals co-evolved towards homeostasis and beneficial relationships^3^. It is important to maintain the proper functioning of host immunity to prevent overexploitation of resources by commensals while maintaining immune tolerance against innocuous stimulation for healthy and beneficial relationships^4^. Environmental incursions such as diet, antibiotics, or changes in geography can alter the gut microbiome leading to impairment in the human-microbiome relationship. Diseases like rheumatoid arthritis (RA), metabolic syndrome, celiac diseases, malignancies, inflammatory bowel disease (IBD), and neurodegenerative disorders occur due to alterations in the microbiome population^5^. The cross-talk between the immune system and gut microbiome is dynamic, context-dependent, and complex^5^.

Recent findings have demonstrated that the age-mediated inflamed microenvironment enhances autoimmune and inflammatory responses with decreased protective immune responses^6^. Further, there is a decline in immune responses to infection, vaccination, phagocytic activity, antigen capturing capacity, and presentation^7^. With the advancement of age, it has been suggested that there is the possibility of gaining nonspecific innate immunity with a weakening in adaptive immune responses^8^. The professional APCs called dendritic cells (DCs) are vital regulators of immune responses against infectious agents^8^. DCs have the property of both the activation and the induction of tolerance to antigens (self/innocuous)^8,9^. The DC maturation and activation depend on antigen uptake, processing, and the delivery of the pathogen’s danger signals^8^. The differentiation and activation of naive T cells can only be done by DCs^10^. The DCs express the optimum levels of MHCs and costimulatory molecules to activate naive T cells^11^. However, the tolerogenic DCs display a low range of costimulatory molecules and proinflammatory cytokines in contrast to immunogenic DCs. Tolerogenic DCs express high amounts of inhibitory molecules like CTLA-4, Lag-3, Tim-3, and PDL-1^8^. The DCs maintain tolerogenic behavior by inducing anergy and clonal deletion of T-cells and the generation of Tregs^12^. Additionally, they are crucial in maintaining peripheral tolerance against self-antigens^13^.

Immunosenescence is an age-associated immune system dysfunction characterized by alterations in several aspects of the immunity, such as loss of adaptive immune diversity and thymic involution. The major characteristics of immunosenescence are the loss of the ability to recognize antigens, a decrease in memory T cells, and persistent low-grade inflammation called inflammaging. The other features of immunosenescence are phenotypical alterations in several immune cell types. Several viruses, such as Epstein-Barr and human cytomegalovirus, influence the immune system, resulting in immunosenescence^8^. Agingaffects differently various subsets of DCs (plasmacytoid DCs, myeloid DCs, follicular DCs). Plasmacytoid DCs (pDCs) are known for their role in initiating an immune response to viral infection and are reported to be majorly affected by aging^14–16^. The gut microbiota plays a significant role in inflammation by producing inflammatory mediators^17^. Although there is not much evidence about the role of DCs in inflammaging, different subsets of DCs present in the gut have been reported to express pattern recognition receptors and respond to microbial products to produce inflammatory cytokines and induction of inflammatory cells^18–21^.

DCs initiate adaptive immunity and process antigens in the draining lymph nodes (DLNs). In mouse models of respiratory virus infections, the DCs in the lung demonstrated decreased migration to DLNs, resulting in decline in T-cell responses because of the age-dependent increase in prostaglandin D2 in lungs^8,22^. It is reported that during aging, there is an increased production of auto-antibodies against self-antigens, and the DCs from the elderly revealed increased reactivity toward human DNA, causing the increase in the production of inflammatory markers and T cell proliferation^23^. The state of chronic inflammation during aging might be an underlying cause of various diseases and deaths that are associated with aging. Although the mechanisms that mediate chronic inflammation associated with aging are not well understood^23^. Future investigation should focus on understanding the chronic inflammatory states in elderly subjects.

The gut microbiome has been observed to be increasingly involved in the immune system’s development, maturation, and maintenance^24^. The microbiome induces peripheral tolerance by the induction of Tregs, tolerogenic DCs, and IgA-secreting B-cells^5^. Advancement in age is associated with alteration in the microbiome, provoking several autoimmune diseases^25^. Chronic inflammation and immune dysregulation persist with the loss of *Firmicutes* and *Bacteroides* and an increase in the *Proteobacteria* population in the gut^26^. It is still unclear how gut dysbiosis and loss of DC tolerance occur with the advancement of age. With a focus on the latest advancements, this article sheds light on the intricate interplay between aging, gut dysbiosis, and DCs, revealing their significant impact on overall health and immune responses.

## Alteration in the microbiome with aging

Microbiome alteration occurs throughout human life and plays an important role in health and well-being^27^. Microbiome changes with age, antibiotics use and the prevalence of diseases. DCs balance the activation and inhibition of immune responses, a unique feature of their functionality. With aging, a gradual loss of DCs tolerance is demonstrated by low expression of costimulatory molecules and proinflammatory cytokines, the deterioration in phagocytic activity, and the inability to induce Tregs, leading to increased chances of autoimmune and inflammatory disorders^28^. The main reason behind the DC tolerance is the enhanced proinflammatory responses and activation of NF-κB^28^. However, the mechanism regarding age-associated DC tolerance is not clear.The studies are required to find out the reasons behind this phenomenon. Contradictory observations have been reported on the differences in the microbiome between young and old subjects, especially the presence of *Bifidobacterium, Ruminococcus*, and *Bacteroides*^27^.

In addition to alterations in the microbiome, dietary factors play a pivotal role in maintaining the homeostasis of the immune system. Research has yielded conflicting findings regarding the impact of specific dietary components, such as carbohydrates, proteins, fatty acids, and phytochemicals, on the aging immune system. These components can exhibit both detrimental and beneficial effects on immune function. When it comes to fatty acids, their influence on pro-inflammatory markers in DCs hinges primarily on the type of fatty acid employed^29,30^. Some studies have suggested that the elderly residing in Mediterranean (MED) regions often enjoy good health, which is frequently attributed to their adherence to MED diets. Further, it has been reported that a MED diet could potentially enhance immune responses in elderly individuals, particularly by positively affecting DC function^29^. The study, involving 120 elderly subjects over the age of 60, revealed that intervention with the MED diet had the potential to mitigate age-associated increase in the secretion of resistin, a marker associated with aging^29^. These findings emphasize the essential role of diet in immune system function and the importance of identifying suitable dietary choices for the elderly to prevent undesirable immune reactions. Further investigation into the potential beneficial effects of the MED diet in larger cohorts across various regions, especially among the elderly, is warranted.

Reports indicating the significant impact of diet on gut microbiota suggest that elderly individuals (aged >65) often exhibit altered dietary patterns, leading to decreased levels of *Firmicutes* and *Bifidobacterium*, as well as an increased abundance of Clostridium and *Bacteroides* ^31,32^. However, it is important to note that these findings may not universally apply across different regions worldwide due to the substantial heterogeneity in dietary habits. This summary aims to outline the role of diets that promote gut health. For instance, the MED diet, primarily characterized by high consumption of fruits, vegetables, whole grains, and legumes, and reduced intake of meat, fish and lactose-rich products has been associated with beneficial effects on gut microbiota. It enhances gut diversity by increasing *Bacteroides* and *Firmicutes* while reducing *Clostridium*^33^. An oriental diet rich in soy protein has also been reported to have a positive impact on the gut flora, particularly by promoting *Bacteroides, Proteobacteria, Bifidobacterium* and *Enterococcus*, while reducing the presence of *Firmicutes* and *Lactococcus*^34^. Furthermore, recent studies have highlighted the influence of specific dietary components, such as fibers, saturated fats and polyphenols on gut microbiota composition. Fiber-rich foods, for instance, have been found to enrich *Bifidobacterium* and *Lactobacillus* while reducing pathogenic colonies^35^. On the other hand, saturated fats tend to increase *Firmicutes* and *Proteobacteria* but decrease *Bacteroides*^36^. Polyphenols, on the other hand, have been associated with a positive impact on *Bifidobacterium* abundance while decreasing *Firmicutes* and *Clostridium* ^37.^

A study in Italy reported that with aging, a different microbiota population of *Lachnospiraceae, Bacteroidaceae*, and *Ruminococcaceae* families dominate, which usually decreases as age advances^38^. A study from China reports a negative correlation between aging and the persistence of *Faecalibacterium*, *Roseburia, and Coprococcus* genera^39^. The human body is densely populated with *Archaea, Eukarya, Bacteria*, and viruses, in which four bacterial phyla of *Firmicutes*, *Proteobacteria, Bacteroides*, and *Actinobacteria* contribute to 98% of the microorganisms^26^.

The microbiota produces short-chain fatty acids (SCFA) from the undigestible fibers^40^. The secondary bile acids (BAs) modulated by the gut microbiome play an essential role in host metabolism and energy balance, primarily through their interactions with nuclear receptors and G protein-coupled receptors (GPCRs). It has been reported that BAs also exert an influence on the composition of the gut microbiome. Moreover, emerging evidence suggests that BAs are of critical importance in the regulation of immune responses *via* their interactions with nuclear receptors and GPCRs. Notably, studies have proposed that the restoration of gut BAs can alleviate experimental autoimmune uveitis (EAU) in animal models, primarily through the suppression of NF-κB-associated inflammatory cytokines in DCs^41^. Furthermore, investigations have demonstrated that DCs treated with BAs exhibit reduced levels of IL-12 and TNF-α in response to bacterial antigens and stimulation. Additionally, BAs induce the differentiation of IL-12 hypo-producing DCs from monocytes through the TGR5/cAMP signaling pathway^42^. These findings collectively suggest that the loss of BAs may lead to reduced DC tolerance and contribute to developing autoimmune and inflammatory diseases. Notably, the restoration of BAs has shown promising results, underscoring the requirement for optimal BA levels in maintaining proper immune system function^41^.

In addition to SCFAs, the gut microbiota abundantly produces various other metabolites, such as LPS, butyrate, propionate, secondary bile acids (e.g., deoxycholic acid), and protein metabolites (e.g., p-cresol sulfate, spermidine, spermine). These metabolites have been shown to impact the immune compartment, particularly DCs. Butyrate, propionate and deoxycholic acid have been demonstrated to inhibit DC development by downregulating the expression of costimulatory molecules and pro-inflammatory cytokines, reducing chemokine expression, and promoting the induction of Tr1 and Tregs^41–45^. The LPS produced by the gut microbiota exhibits pleiotropic effects on the immune compartment. Gut-derived LPS has been reported to enhance the migratory capability of DCs, while p-cresol sulfate has been found to impede DC migration during airway inflammation^46,47^. Another metabolite, spermidine, exerts an immune-suppressive effect by enhancing the activation of indoleamine 2,3-dioxygenase (IDO), p-Src, and FOXO3, while inhibiting NF-κB activation when co-cultured with DCs^48,49^.

Microbiota plays an essential role in food digestion throughout the alimentary canal. In addition, the digestive slurry gets mixed with microbiota for synthesizing, absorbing, and extracting metabolites and nutrients^40^. The functions of SCFA are diverse such as regulating immune cells (activation of CD8^+^ T and differentiation of CD4^+^ T cells), controlling microbial functions, maintaining intestinal integrity, microbial energy source, and combating pathogens^26^.

The studies on the microbiome can be classified into two categories i) alterations in gut microbiota composition with age; ii) changes in the microbiome along with aging disorders (Table 1). From these two categories, the major finding is literature-based recognition of specific groups of taxa that demonstrate changes with aging, whether healthy or unhealthy^50^.

**Table 1.** Changes in the microbiome of aged subjects related to the aging disorder.

Changes in the microbiome along with the aging disorder		Disorder	Country	References
Increase	Decrease			
An increase in the population of *Lactobacillus* and Pathobionts	–	Reduced bone mass density	Ireland	^50^
An increase in the population of *Anaerotruncus*, *Coprobacillus*, and *Parabacteroides*	Major SCFA-secreting bacteria and main gut microbiota	Frailty		
*Parabacteroides*, *Anaerotruncus*, *Coprobacillus*				
Pathobionts				
	SCFA producer *Faecalibacterium prausnitzii*		United Kingdom	
	*Prevotella copri*, SCFA secreters, *Coprococcus eutactus*		Korea	
Pathobionts, *Ruminococcus*, *Coprobacillus*	Alpha diversity		United States	
Atopobiaceae, *Ruminococcus torques*	*Eubacterium, Gemella*, *Azospira*. *ruminatium*		China	
*Ruminococcus*	Christensenellaceae, *Barnesiellaceae*		Italy	
Pathobionts, *Ruminococcus*, *Lactobacillus*, *Blautia*	*Prevotella*, *Odoribacter*, Christensenellaceae, *Barnesiella*, *Butyricimonas*, *Lachnospira*, alpha diversity	Cognitive decline	United Kingdom	
NA	*Akkermansia* and Lentisphaerae		United States	
NA	*Akkermansia*			
*Lactobacillus and* Pathobionts	*Faecalibacterium, Roseburia, and Prevotella*	Chronic kidney disease and frailty	Italy	
*Coprobacillus*, *Eggerthella*, *Anaerotruncus*, *Megasphaera*	*Cetobacterium*, *Faecalibacterium*, Lachnospiraceae, *Prevotella*	Reduced physical activity	United States	
NA	*Bilophila* positive. *Faecalibacterium prausnitzii*		Sweden	
*Lactobacillus and* Pathobionts	*Roseburia*, *Faecalibacterium*, and *Prevotella*	Chronic kidney disease and frailty	Italy	
*Clostridioides difficile*	*Oscillospira*	Cardiometabolic disease	Japan	
Pathobionts	*Prevotella*, core SCFA producers, *Bifidobacterium*, *Odoribacter*, *Victivalis*	Parkinson disease	Germany	
NA	*Roseburia*, *Bifidobacterium*, and *Lactobacillus*	Reduced bone mass density	China	
*Collinsella*, *Bifidobacterium, Paraprevotella*	*Akkermansia*, *Faecalibacterium*, *Prevotella*	Obesity and metabolic syndrome	Ireland	
Pathobionts	*Butyrivibrio*, core SCFA producers, and *Adlercreutzia equolifaciens*	Alzheimer disease	United States	
	*Oscillospira*, Christensenellaceae, Ruminococcaceae, Lachnospiraceae	Visceral fat deposition	United Kingdom	
	SCFA secreting bacteria, *Bifidobacterium adolescentis*, *Butyrivibrio* other SCFA secreters	Migraine		
Xenobiotic degradation-pathway-genes and Pathobionts	NA	Comorbidities (among long-living individuals)	China	
NA	*Butyrivibrio crossotus*, *Alistipes* sp., *Bacteroides* sp., *Prevotella stercorea, Akkermansia muciniphila*	Mortality (among centenarians)	China	
Pathobionts	*Akkermansia*	Comorbidity	United States	
NA		Progeria	Spain	

Pathobionts include the following lineages: Bacteroides fragilis, Campylobacter Streptococcus, Fusobacterium, Parvimonas, Actinomyces, Campylobacter, Porphyromonas, Anaerotruncus, Staphylococcus, Flavonifractor, C. hathewayi, C. bolteae, C. citroniae, C. clostridioforme, C. symbiosum, C. hylemonae, C. scindens and C. difficile. Ruminococcus torques, R. gnavus Corynebacterium, Desulfovibrio, Bilophila, Eggerthella, all Enterobacteriaceae, Clostridium asparagiforme and SCFA secreters include: Roseburia, Coprococcus, Eubacterium, Faecalibacterium, Dorea, and Blautia. NA, not available.

Recent studies reported the relative differences in the population of *Clostridium cluster XIVa, Escherichia, Shigella, Blautia, Faecalibacterium, Ruminococcaceae, Lachnospiraceae*, and *Erysipelotrichaceae* in aged subjects of more than 100 years^26^. Some investigations suggested that the gut gets enriched with *Proteobacteria* and *Bacteroidetes* and drops in *Lactobacilli* and *Bifidobacteria* as age advances. Understanding these age-related changes may inspire targeted interventions for healthy aging and disease prevention. It was revealed that the *Bifidobacteria* adhered more to the intestinal mucosa in infants and young adults than in old subjects^51^. Such studies reveal that the aged subjects have less ability to adhere and colonize *Bifidobacteria*. The intestinal mucosa may lose adhesion property (decay in adhesion proteins), which might be the reason behind the failure in colonization. The *Bifidobacteria*, which are more sensitive to changes in the intestinal mucosa adhesion property (decreased affinity), may be lost, although the exact mechanism is yet to be identified. *Bifidobacteria* use lipoteichoic acids (LTA) for adhesion to the intestinal mucosa^52^. The different *Bifidobacteria* groups might express LTA to different levels, and other microorganisms may compete with it for adhesion to the intestinal mucosa. Probiotic *Bifidobacterium* having good adhesive characteristics might help elderly subjects with their colonization in the gut. *Akkermansia, Christensenellaceae, Oscillospira*, and *Bifidobacterium* are health-associated bacteria^38^.

*Christensenellaceae* and *Oscillospira* have been suggested to control leanness and decrease inflammatory disorders in elderly subjects^53^. *Akkermansia muciniphila* has been demonstrated to control metabolic and inflammatory diseases, protect epithelial integrity, and support SCFA-secreting bacteria^54^. SCFA and lactate produced by *Bifidobacterium* help reduce inflammatory microbes^26^. A study on 371 subjects comprising newborn babies and centenarians revealed an aging-associated increase of *Oscillospira* compared to middle-aged adults and children. Some beneficial bacteria are lost while advancing with age progression^55^. The microbiome alteration occurs in old age despite the influence of external environmental factors such as medications, sedentary lifestyles, diet, exercise, inter-individual variation, and geographical locations.

## Influence of gut-dysbiosis on DCs

The DCs play an important role in tolerance by acting as a connecting bridge between adaptive and innate immunity^7^. The tolerogenic DCs control the Tregs and effector T cell responses^56^. The function of DCs, particularly regulatory, gets impaired as age advances and with the loss of beneficial gut microbiota^7^. The unique characteristic of DCs is the induction of Th1 response in the presence of infection or Tregs in the absence of any infections. The CD103^+^ DCs in mice regulate the balance between Th1 cells and Tregs via the p38-MAPK signaling pathway during their differentiation from naïve T cells^57^. The DCs may change the T cell phenotype by alteration of danger signals, recruitment of inflammatory cytokines, or changed conditioning. The gut microbes use LTA/polysaccharides or DNA to cross-talk with the intestinal mucosa and produce antibacterial effects against pathogenic microorganisms by conjugated linoleic acid and bacteriocins^58^.

Microorganisms can modulate the function of DCs by interacting with the toll-like receptors (TLRs) expressed by DCs^59^. The different microorganisms network differently with DCs through distinct TLRs^60^. The DCs (CD10^+^ CD11b^+^) expressing TLR5 cross-talk with bacterial flagellin to induce Th17 cells^59^. The production and development of the Th17 cell anti-microbial peptide RegIIIγ immune response depend on activating the DCs that produce IL-23 and IL-6^18^. The DCs can also be activated via TLR7 agonist to generate CD8^+^ T cell responses *in vivo*^61,62^. The gut microbiota co-evolved with the host, which renders the APCs to protect from infectious agents while maintaining self-tolerance with gut microorganisms. The best example of tolerance is that the DCs of the spleen produce low levels of IL-10, whereas the DCs of Peyer’s patches secrete more IL-10 under similar conditions^63^. There is a pivotal role of gut microorganisms in regulating the development and maturation of APCs, particularly DCs. Research studies revealed that in the germ-free (GF) mice, the DCs population decreased but not in the systemic circulation. Interestingly, gut colonization with *Escherichia coli* in the GF mice recruited the DCs to the intestines^64^. DCs expressing CXCR1 and CD70 get activated through microbe-derived ATP, which induces Th17 cells^65^.

The microbial metabolites influence the DC’s development by altering bone marrow (BM) hematopoiesis, which changes the type/phenotype of DCs in the airways and lungs. The DC number in lymph nodes and spleen remains unchanged in germ-free mice compared to specific pathogen-free mice (SPFM)^66^. The studies conclude that microbiota dysbiosis does not impact the steady-state generation of DCs. This does not suggest that the gut microbiota does not affect DCs^66^. The treatment of SCFAs derived from microbiota has demonstrated the generation of DC progenitors and influences their development^66^. From the findings, it is evident that gut dysbiosis has a negative influence on the DCs functions. The maintenance of DC tolerance is crucial for homeostasis. It is important to find microbial species and their metabolites that can recover the loss of DC tolerance. As the microbiome is diverse with many organisms, sophisticated methods should be inculcated to identify the species responsible for DC tolerance and maintenance. In the case of lung disorders such as COPD, infections, and cystic fibrosis, the DCs play a crucial role. *Saccharomyces, Lactobacillus*, and *Bifidobacterium* are the most commonly used probiotics as supplements in infectious diseases, IBD, and colon cancer. Probiotics act as immunomodulatory agents that activate the defense pathways in various respiratory disorders^67^.

The development of probiotic formulations will help in managing old-aged subjects suffering from lung disorders. The involvement of microbial metabolites and their functionality in various lung disorders is paramount such as SCFA metabolites have proven beneficial effects in treating several lung diseases. The susceptibility to lung disorders due to gut dysbiosis leads to a diminished production of SCFAs. With the advancement in research findings, SCFA producers could be formulated as probiotic supplements for managing lung disorders. Future investigations should focus on whether SCFA could be formulated for sustained release in lung disorders. The probiotics have shown promising results in stimulating the DC regulatory functions by targeting specific pathogen recognition receptors (PRR) and signaling pathways^68^. Such results are not only for immune functionality but can be interventional in inflammatory bowel disease^68^. Such studies show promise in potentially restoring the impaired tolerogenic behavior of DCs due to aging and gut dysbiosis. Given that aging and gut dysbiosis can trigger autoimmunity, further investigation into probiotic treatments is warranted, particularly in the context of autoimmune and lung disorders.

## DCs dysfunction with age

Aging leads to inflammation, autoimmunity, immunodeficiency, infection susceptibility, and weak/non-response to vaccines^15^. This suggests that the immune response against infectious agents decreases during aging, and immune reactivity against endogenous molecules becomes more prominent because of increased inflammatory response. Further, the decrease in immune tolerance and loss of tissue integrity gives rise to new antigens and autoimmune immune reactivity through molecular mimicry^69^. The continuous impairment in the functions of immune cells in aged subjects compared to young subjects are the major reason for morbidity and mortality.

With significant advancements in molecular and cellular mechanisms in science, there is still much to unravel concerning the changes associated with aging-mediated immune dysfunction and chronic inflammation. A recent study reported that age-related impairment of DC function leads to immune dysfunction, such as a loss of DC tolerance and increased chronic inflammation^70^. DCs express pathogen-sensing receptors like Toll-like receptors (TLRs), C-type lectin receptors (CLRs), and NOD-like receptors (NLRs). A comparison between young and elderly populations revealed a decrease in the expression of TLR1, TLR2, and TLR8 in classical DCs (cDCs) among the elderly, while TLR2 expression remained unchanged. Additionally, TLR7 was downregulated, with no change observed in TLR9 expression in plasmacytoid DCs (pDCs) in aged individuals^71^. Another group discovered that both TLR7 and TLR9 expressions were decreased in pDCs, leading to a selective influence on the declining percentage of pDCs during healthy aging, while no association was found with any alteration in myeloid DCs (mDCs)^72^. Interestingly, a study involving children showed that the number of pDCs decreased by 2.5-fold in the first 10 years of life, while mDCs remained unaffected^73^. These findings suggest that specific types of cells undergo alterations during aging, warranting further investigation to understand this phenomenon in greater depth^73^. However, there have been contrasting results published regarding pDCs and aging. While some studies have demonstrated a decline in the number of pDCs with age^72,74,75^, other investigations claim no change in pDC levels^76^. It is essential to conduct more comprehensive research to unravel the intricacies of age-mediated immune dysfunction and its impact on DCs. Further studies should focus on understanding the underlying mechanisms responsible for the observed changes in different types of DCs during the aging process, potentially leading to new therapeutic interventions targeting age-related immune dysfunction and inflammation.

The maintenance of self-tolerance is a crucial function of DCs. These cells are consistently exposed to antigens produced from damaged tissues and dead cells. Although the DCs take up these self-antigens, they remain inactive and express low levels of costimulatory markers. Consequently, they fail to present the antigens to T cells^28^. Presenting self-antigens to T cells without costimulatory signals results in T cell anergy, a state of unresponsiveness. However, during inflammation or tissue injury, the DCs may become activated and present self-antigens along with costimulatory signals to T cells, leading to autoimmunity. Aging contributes to impaired function, loss of homeostasis, and increased susceptibility to death. Aging reduces DC tolerance, leading to gut dysbiosis and inflammation. This impacts mental well-being and heart health and causes tissue damage. Lung and autoimmune disorders also become more common with aging and gut dysbiosis. Understanding and addressing these changes is vital for healthy aging. The elevated basal level of NF-κB in the DCs of the elderly indicates that these DCs are in an activated state^28^. Similarly, we observed an increased level of NF-κB in the DCs of old mice compared to young mice^7^. After apoptosis, self-DNA is released and phagocytosed by DCs. In the elderly, DCs have a reduced capacity to uptake apoptotic cells, leading to defective clearance and immune responses instead of tolerance against self-antigens^28^.

During viral pneumonia, DCs fail to migrate into aged lungs and from lungs to lymph nodes, rendering them unable to prime naïve T cells against the influenza virus^77^. Reports suggest age-related defects in DCs presenting antigens *via* MHCI to prime CD8^+^ T cells, with diminished production of IL-1β and inflammasome activation^78^. The impact of antibiotics on lung microbiota is not well-studied, although it has been established that antibiotics alter the microbiome and lung functions in cystic fibrosis^79^. Some studies indicate that antibiotics increase the death rate in animals infected with *S. pneumoniae* and Influenza A virus^80^. The influence of antibiotics and age-related gut dysbiosis on DC function requires thorough examination, and appropriate probiotic preparations should be investigated in disease models. Supplementation of depleted beneficial bacteria with age through diet is essential for maintaining homeostasis. Recent research has shown that bacteriotherapy holds promise against frailty and unhealthy aging^81^. External factors such as drug therapy, exercise, and diet play crucial roles in healthy aging and increasing life expectancy^81^. Moreover, the intestinal microbiota is considered a major factor in the anti-aging process^81^. Future studies should focus on elucidating the mechanisms involved in the anti-aging process for a better understanding. The elderly population is an important part of society and is more susceptible to various diseases. Therefore, the scientific community needs to investigate better therapies that can improve the quality of life during the later stages of life.

## The modulation in the function of DCs in the aged population

Dendritic Cells (DCs) in the infected aged community have been found to weakly induce Tregs^82^. Aged individuals are more susceptible to infections caused by *Chlamydia, pneumonia* and *influenza*^83^. The TLR function of pDCs and mDCs becomes impaired with age more than 65 years compared to younger individuals of age between 21–30 years^71^. This impairment is associated with a poor Ab response against influenza viruses^83^. Moreover, aged individuals are more prone to chronic obstructive pulmonary diseases such as bronchitis, emphysema, and asthma. Reduced DC tolerance in the airways allows for the invasion of pathogenic agents and inflammation, increasing susceptibility to lung-associated diseases. Unstimulated DCs from aged subjects exhibit increased expression of cytokines and costimulatory molecules, resulting in heightened epithelial permeability^28^. As a consequence, the functionality of other cells is compromised, leading to the activation of T cells without any infection and similar reactions may occur in the skin and gut. Infections on the airway, gut, and skin surface are more common in aged subjects due to the loss of integrity of cell-to-cell junctions^84^.

The gut microbiome synthesizes Short-Chain Fatty Acids (SCFA), such as butyrate, acetate, and propionate, which are crucial metabolites for normal body homeostasis. The percentage of SCFA declines in aged subjects compared to young subjects^85^. SCFA induces Tregs, prevents DC activation, and maintains DC tolerance in the gut^86^. DCs sense SCFA metabolites through special receptors like GPCRs (GPR109A, GPR41, and GPR43), which have different affinities and specificity toward particular metabolites^86^. Several immune cells, including Tregs, neutrophils, and macrophages, express GPR43 and GPR109A, which play roles in regulating microbiome homeostasis by binding to butyrate and nicotinic acid, acting as anti-inflammatory molecules^87^. Studies also suggest that propionate and GPR41, a receptor for SCFA, play a crucial role in generating DC and macrophage precursors^88^.

Infections caused by *Clostridium difficile* and *Helicobacter pylori* in the gut are dangerous in aged subjects, leading to hospitalization^89^. As people age advances, microbiome alteration occurs in parallel, with an increase in *Enterobacteriaceae*, Gram-negative, and other pathogenic bacteria in the gut^89^. The LPS secreted by Gram-negative bacteria activates APCs like DCs and macrophages, leading to inflammation^70^. The increase in LPS-secreting Gram-negative bacteria is one of the reasons behind inflammation in aged subjects. Therefore, appropriate probiotics should be supplemented in elderly subjects to balance the healthy gut microbiome population and avoid inflammatory reactions.

Infections such as SARS-CoV-2 and influenza impair APCs, TLRs, cytokines, and T cells, resulting in poor immune responses^90^. Age-mediated gut dysbiosis weakens the function of DCs and T cells, making old-aged subjects susceptible to lung infections. The supplement of probiotics regulates immune responses via modulation of the TLR signaling pathway, thereby regulating NK cells, DCs, and Th1 and Th2 immune responses^90^. Probiotics have demonstrated preventive effects on upper respiratory infections, improved outcomes, and reduced disease duration and severity in children and adults^91^.

Numerous probiotics have been investigated in clinical trials in elderly subjects to improve immune responses against influenza vaccination and infections. *L. plantarum* (CECT7315/7316) has shown an immuno-stimulating effect and might improve immune responses against the influenza virus in old subjects^92^. Additionally, two randomized clinical trials have revealed that daily administration of specific probiotic preparations enhances specific Ab responses against influenza vaccination in old-aged subjects (70 years old), suggesting potential beneficial effects for managing lung infections^93^. However, evidence suggests that not all probiotic preparations may improve protection against respiratory infections^90^. Some investigations have shown no significant effect of probiotics in decreasing respiratory infections^94^. For instance, a study focused on 737 healthy old subjects reported that administration of *Lactobacillus casei* for 176 days did not improve respiratory symptoms or show positive immune responses in vaccinated individuals compared to controls^95^. Therefore, it is crucial to identify bacterial species that can reverse regulatory DC functions and prevent the occurrence of diseases in aged subjects.

## Molecular mechanisms in DC dysfunction during aging

**Figure 1.**
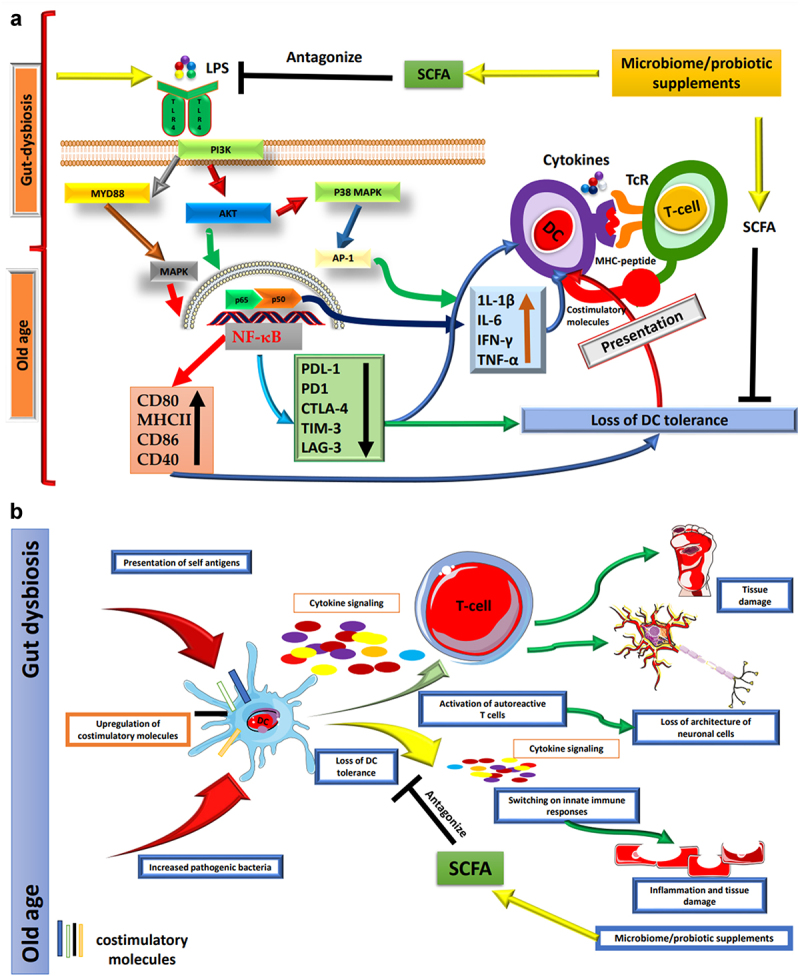
(a) Age-related changes in the gut microbiota affect DCs. As individuals progress through the aging process, significant alterations occur within the gut microbiome. These changes are characterized by an augmented presence of Gram-negative bacteria, which, in turn, leads to an increased secretion of LPS. The LPS exerts its influence via TLR4 on DCs, thus initiating a complex signaling cascade. This cascade involves critical components such as MYD88, AKT, PI3K, and MAPK pathways, ultimately culminating in the activation of the transcription factor p65 and a subsequent elevation in the basal activity of NF-κB. The heightened activation of NF-κB within DCs results in a noteworthy upregulation of proinflammatory cytokines and the augmentation of essential costimulatory molecules, including CD80, CD86, MHCII, and CD40. Concurrently, this activation leads to the downregulation of immune checkpoint proteins. These activated DCs then proceed to present antigens bound to MHC molecules and engage in interactions with T cells through the TCR, thus facilitating immune responses. This shift in the equilibrium of immune signaling makes DCs more inclined to mount vigorous inflammatory responses. Consequently, these activated DCs evolve into proficient antigen captors and presenters to T cells, thereby promoting immune responses against potential threats. However, this heightened state of immune activation also brings about a potential loss of DC tolerance, rendering the immune system more susceptible to autoimmune reactions and the development of chronic inflammatory diseases. The maintenance of a harmonious and balanced immune response is paramount for healthy aging, as the dysregulation of DC function can significantly contribute to a spectrum of age-related pathologies. One promising approach to ameliorate the adverse impacts of gut dysbiosis on DCs involves the supplementation of SCFAs. SCFAs are microbial metabolites generated through the fermentation of dietary fibers within the gut. These compounds have been demonstrated to antagonize TLR inflammatory pathways, thereby offering a potential means to safeguard DC tolerance and sustain a more balanced and harmonious immune response. (b) Aging induces various changes in the human body. Throughout the aging process, the human body undergoes a *multitude of* transformations, encompassing significant shifts within the gut microbiome. These alterations wield a profound influence on the immune system, with a particular emphasis on DCs, pivotal regulators of immune tolerance. DCs play a critical role in the recognition and presentation of antigens to T cells, thus regulating immune responses. The sustained activation of DCs in the elderly populace can precipitate adverse consequences, given that these cells progressively lose their ability to uphold immune tolerance toward self-antigens. Consequently, this loss of tolerance manifests as chronic inflammation, autoimmunity, and tissue damage. The unrestrained expression of costimulatory molecules and the presentation of self-antigens by DCs to autoreactive T cells usher in a disarray of immune responses within aging individuals, fostering inflammation, disruption of neuronal cell architecture, and tissue damage associated autoimmune diseases. It is worth noting that age-related maladies can potentially be ameliorated through the judicious supplementation of probiotics and SCFAs. Probiotics, being beneficial microorganisms, can reinstate equilibrium within the gut microbiome and foster the tolerogenic behavior of DCs. By modulating the functions of DCs, probiotics hold the promise of mitigating inflammation and enhancing immune tolerance, thereby opening a promising avenue for the management of age-related health conditions. The artwork used in this figure was adapted from Servier Medical Art (http://servier.com/Powerpoint-image-bank). Servier Medical Art by Servier is licensed under aCreative Commons Attribution 3.0 Unported License.

Molecular mechanisms underlying DC dysfunction during aging are not fully understood, but NF-κB signaling plays a crucial role. NF-κB, the master regulator of inflammation, is implicated in various diseases, including autoimmunity and inflammatory disorders^96,97^. Overexpression of NF-κB subunit RelA/p65 induces a senescent phenotype in cells, while low expression delays age-associated diseases in *in vivo* models (Figure 1(a)^98,99^. Our group reported that age and gut dysbiosis lead to increased p65 phosphorylation in murine models, and *Lactobacillus planetarium* supplementation can prevent overt NF-κB activation, which is associated with DC maturation^7^. Other studies also demonstrate increased basal activation of NF-κB/PI3K in monocyte derived dendritic cells (MODC) and circulatory DCs^23,71^. The exact cause of DC dysfunction, whether external or intrinsic, remains unclear. Age-associated increases in prostaglandins and pro-inflammatory cytokines in circulation can trigger DC maturation and cytokine secretion even in the absence of disease^100,101^. Additionally, an increase in bone marrow fat levels in old age may contribute to cytokine release, further promoting DC maturation and activation^100,101^.

Epigenetic modifications, including DNA methylation and chromatin alterations, can also impair DCs during the aging process. In older individuals, inhibitory H3K9 histone proteins exhibit an increased binding affinity to IFN type I and III promoters in myeloid DCs. This leads to a reduction in IFNs production, particularly in the context of viral infections such as influenza^102^. Further investigation is essential to delve into intrinsic alterations within DCs in elderly subjects. This exploration should encompass the examination of factors like miRNAs, epigenetic markers, and various signaling pathways (such as mTOR/PI3K/PTEN/Hedgehog/NrF2/Wnt) to gain a comprehensive understanding of molecular-level changes.

Microbial metabolites influence DCs through various pathways, altering bone marrow hematopoiesis and influencing DC development in different body parts^88^. Probiotics, like the VSL3 preparation containing specific bacterial strains, have shown promising results in ulcerative colitis patients^103^. The protective activity was mediated through inhibition of the PI3K/Akt and NF-κB axis, leading to reduced pro-inflammatory cytokines and increased anti-inflammatory cytokines in colonic tissue^104^.

Probiotics have demonstrated the potential to restore DC functionality. The VSL3 probiotic preparation showed protective effects against wild-type colitis but not in TLR9 knockout mice, indicating that gut microbiota acts through specific TLR receptors on DCs or other APCs^105^. Bacterial metabolites and pathogen-associated molecular patterns (PAMPs) often act via TLR pathways, which are interlinked with inflammatory signaling cascades. Dysbiosis in the gut increases inflammatory signaling, resulting in the loss of immune tolerance in DCs. SCFA metabolites, on the other hand, can antagonize TLR4 signaling, offering probable future therapies for various inflammatory conditions. Research should now focus on elucidating the pathways of microbiome metabolites and evaluating their therapeutic potential against lung-associated diseases.

## Influence of gut microbiota dysbiosis on the health of aged subjects

Hippocrates (460–370 BCE), the father of modern medicine, proposed that “All Diseases Begin in the Gut,” a concept still relevant today^106^. Gut dysbiosis, a natural occurrence in old subjects, can lead to various serious consequences, including loss of DC tolerance, susceptibility to infections, reduced bone mass index, neurodegenerative diseases, autoimmune diseases, and inflammatory diseases^26^.

Clinical studies using fecal samples have investigated gut dysbiosis in young (28–46 years) and old (>65 years) subjects. In old subjects, the phylum *Firmicutes* was dominant at 40%, and phylum *Bacteroidetes* was at 57%, whereas in young subjects, *Firmicutes* was 51% dominant, and *Bacteroidetes* was 41%^106^. Another investigation reported a decline in the *Firmicutes/Bacteroidetes* ratio from 10.9 to 0.6 between young (25–45 years) and old (70–90 years) subjects^107^. The ratio of *Firmicutes* and *Bacteroidetes* was higher (9-fold) in old mice/rats than in young animals, with the old animals demonstrating anxiety behaviors and cognitive impairment^108^. The gut-brain axis, linking the gut microbiome to brain functioning, has gained attention, revealing links between the gut microbiome and various body parts in physiological and pathological aspects. Evidence suggests that gut microbiome dysbiosis can influence brain functions and increase the risk of neurological diseases like Alzheimer’s disease, substance use disorders (SUDs), COPD, and Parkinson’s disease^109,110^.

Aging leads to changes in the gut microbiome, increasing Gram-negative bacteria and LPS secretion. This activates DCs through the TLR4 pathway, leading to NF-κB activation and pro-inflammatory cytokine upregulation. DCs become more efficient at presenting antigens to T cells but may lose tolerance, promoting autoimmune reactions and inflammation. SCFA supplementation could help maintain a balanced immune response and mitigate the effects of gut dysbiosis on DCs (Figure 1A). Gut dysbiosis is also being studied in the context of inflammatory bowel disease (IBD). Although pathogenic bacteria were initially proposed as the cause of IBD, current research suggests that gut dysbiosis may play a major role in its pathogenesis^111^. The rising incidence of cancer in older adults may be linked to gut dysbiosis. By 2035, there is expected to be a 60% increase in cancer cases in older adults, with more than 14 million cases^112^. Some bacteria, such as *Bifidobacterium pseudopodium*, *Lactobacillus johnsonii*, and *Olsenella* species, are being explored for their potential in treating colorectal cancer (CRC). Probiotics are also under investigation in clinical trials for managing CRC^113^. Probiotics and immune checkpoint inhibitors are being used to enhance therapeutic potential against various types of cancers.

Aging impacts the gut microbiome and DCs, thereby affecting immune tolerance. Persistent DC activation leads to chronic inflammation and autoimmune diseases. Probiotics and SCFAs offer potential solutions to restore gut balance and promote DC tolerance, managing age-related conditions (Figure 1B). Age-associated lung diseases can be managed with probiotics, exercise, proper diet, and bacterial-associated metabolites. Healthy aging is essential to prevent age-associated diseases, as the elderly population often relies on various therapeutics. Lifestyle factors such as diet, exercise, and water intake play a significant role in disease prevention in old age. Importantly, managing age-associated diseases requires a proactive approach to maintain a healthy lifestyle and prevent the occurrence of these conditions.

## Role of aging in the gut-lung axis

During aging, physiological changes in the lung lead to a decline in lung function capacity and homeostasis. Lung-associated diseases such as tuberculosis, cystic fibrosis, pneumonia, chronic obstructive pulmonary disease (COPD), and pulmonary fibrosis become more prevalent with age^114^. The occurrence of lung disease during aging is influenced by immunological, cellular, and physiological changes. Understanding age-associated alterations in lung physiology is essential for comprehending the healthy aging process^114^. Healthy lung status is considered a predictor of aging, with subjects having healthy lungs living longer compared to those with poor lung functionality who are more prone to diseases such as cardiovascular diseases, diabetes, and cognitive decline^64^.

The nose, gut, and throat microbiota develop coordinately at the young age^115^. The gastrointestinal tract (GIT) and respiratory tract share a common entry of microbes in the oral cavity, leading to significant overlap between the lung and gut ecosystem^116^. The Human Microbiome Project data revealed that 45% of the microbiome population from stools overlaps with the oral cavity^117^. Aging results in a natural decline in lung functions due to genetic and environmental factors. Several hallmarks contribute to changes in the lung aging axis, including genomic instability, loss of proteostasis, epigenetic changes, telomere attrition, mitochondrial dysfunction, stem cell exhaustion, altered intracellular communication, deregulated nutrient sensing, and dysregulation of the extracellular matrix (ECM)^118^. These changes lead to physiological and structural alterations in the lung. Major changes during aging include abnormalities in the structure of cilia, mucins, and antioxidants in the lining of the epithelium, as well as reduced lung muscle strength in the diaphragm, resulting in a weakening in lung functionality and increased susceptibility to pulmonary diseases^114^. In aged lungs, the size increases due to ECM alterations, leading to increased expiratory lung volume (ELV) and decreased elastic recoil^119^. Previous studies have indicated that gut dysbiosis is linked to acute and chronic lung-associated diseases^118^. Recent research reported that 10–18% of SARS-CoV-2 subjects experienced gastrointestinal symptoms, and older people were at an increased risk of developing severe illness^120^. In old-aged subjects, increased epithelium permeability in the lungs and gut suggests impairment in tissue functions^121^.

The presence of high bacterial diversity is a hallmark of a healthy gut microbiota^122^. Reports indicate that subjects with chronic diseases have an increased abundance of harmful bacteria such as *Escherichia* and *Clostridium* species, along with a decrease in commensal bacteria^123^. The mechanisms behind these bacterial population changes are not well understood and require further investigation. For asthma subjects, metagenomic analysis of stool samples revealed decreased microbiome diversity compared to healthy subjects in the United Kingdom^124^. Additionally, SCFA-producing bacteria like *Coprococcus eutactus* and *Faecalibacterium prausnitzii* were reduced, while *Eggerthella lenta* and *Clostridium* species increased in asthma subjects^124^.

In lung diseases like cystic fibrosis (CF), gut dysbiosis and intestinal inflammation are frequently reported due to antibiotic use in both young and old subjects^125^. Antibiotic use can disrupt the gut microbiome and lead to various ailments. The gut-lung axis has been demonstrated in CF with pathogenic colonization in the lungs and a decrease in the beneficial microbiome population. Metagenomics analysis revealed that in CF subjects, *Propionibacterium acnes, Clostridium difficile*, and *Staphylococcus* spp were more abundant in stools than in healthy subjects, while populations of beneficial bacteria like *Roseburia, Anaerostipes, Blautia, Pseudobutyrivibrio, Faecalibacterium, Subdoligranulum, Streptococcus, Dorea*, and *Coprococcus species* were depleted^125^. Considering the microbiota while prescribing antibiotics in clinics is crucial, and certain diets can also impact the lung microbiome, with some spiced foods having negative effects. Major causes of diseases are lack of exercise and improper diet intake. Maintaining a healthy lifestyle is essential to support the body’s natural defenses and promote overall well-being.

Fecal Microbial Transplantation (FMT) has been shown to increase the transcriptional activity of NF-κB in the lungs with lung injury in mouse models, causing an increase in TNF-α, IL-1β, and IL-6 expression, as reported in some studies^126^. However, the transplantation of fecal bacteria prevents the transcriptional activity of NF-κB and decreases the secretion of proinflammatory cytokines^127^. Some studies have demonstrated that FMT restores the gut microbiome diversity and abundance in the mouse model with pneumonia, prevents tissue damage and inflammation, and maintains the balance between Tregs and Th17 cells^126,127^.

The diet and exercise are very important in maintaining homeostasis of body functions. Diet has been found to influence the gut microbiome. The nutrition rich in fibers, like the MED diet, promotes a healthy gut microbiome and releases butyrate^128^. The benefits of a good diet and a healthy gut microbiome help improve lung functions in diseased conditions such as COPD, asthma, or chronic inflammatory conditions^128^. Some clinical studies have shown a significant and independent correlation between low exercise and low adherence to MED diets and changed pulmonary functional patterns^129^.

Even though probiotics were quite a success, a certain drawback has emerged, e.g., transferring antibiotic-resistant genes to other microorganisms in the gut to produce harmful metabolites. Hence, exploring other alternatives like prebiotics, synbiotics (a mixture of probiotics and prebiotics), and fecal FMT becomes necessary. Applying prebiotics (fructans, oligosaccharides, fructooligosaccharides, galactooligosaccharides, lactose) to enhance gut diversity has recently been explored in altering the immune compartment and their implications for various diseases^130^. Using prebiotics, synbiotics, and FMT to enrich gut species (e.g., *Bifidobacterium*) responsible for DC health may be an interesting approach to overcoming the problem of DCs associated with aging.

## Role DCs in age-associated lung diseases

The epithelial lining covering the nasal passages and airways acts as a physical barrier and regulates immune responses against pathogens. DCs and airway epithelial cells (AECs) closely interact, influencing each other’s functions^131^. DCs release interferons during viral infections, enhancing MHCI expression on AECs to combat virus-infected cells^132^. Immune cells infiltrate infection sites through tight epithelial junctions, facilitated by DC-released pro-inflammatory cytokines^133^. AECs also impact DC function, expressing PRR for allergens and initiating pathogen-host interactions^134^.

LPS-mediated TLR4 signaling on AECs induces lung DC migration to mediastinal nodes^134^. House dust mite (HDM) in mouse models triggers AECs to release CCL2 and CCL20, attracting immature DCs/monocytes to the lungs^131^. The AEC-DC interaction is vital for Th2 immune responses against allergens^135^. AECs release TSLP and IL-25/IL-33 upon allergen exposure, acting on DCs through OX40-OX40L cross-talk to elicit Th2 immune responses^135^. IL-25 induces DCs to release CCL-17, contributing to Th9-mediated immune responses during allergic inflammation^136^.

**Figure 2.**
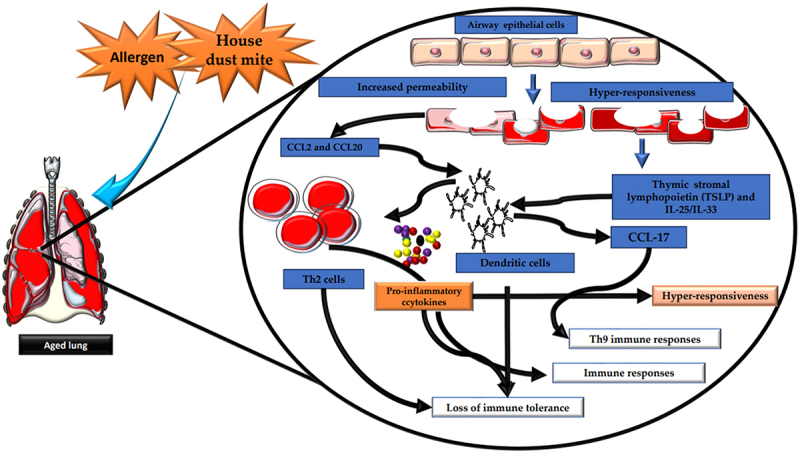
Aging induces significant changes in the structural and functional integrity of the airways. Aging is linked to heightened airway epithelium permeability, rendering it more susceptible to environmental challenges and allergens. This increased vulnerability can induce elevated inflammation and immune reactions within the airways. Airway epithelial cells (AECs) play a pivotal role in sensing and responding to allergens and environmental stimuli. Following allergen exposure, AECs release cytokines, including IL-25, IL-33, and TSLP, which activate DCs. DCs present antigens to T cells, thus initiating and modulating immune responses. In the context of aging, DC activation by AEC-released cytokines can lead to the generation of proinflammatory cytokines, contributing to chronic airway inflammation. Moreover, AECs release chemokines such as CCL2 and CCL20, further activating DCs and promoting the recruitment of inflammatory cells to the airways. This sequence of events can result in the production of CCL17 by DCs, associated with the recruitment and activation of Th2 cells and Th9 cells involved in allergic responses. Dysregulated immune responses in aging airways may lead to diminished immune tolerance, increasing susceptibility to lung tissue damage and heightened antigen hyperresponsiveness, which manifest to asthma or chronic obstructive pulmonary disease (COPD) in older individuals.The artwork used in this figure was adapted from Servier Medical Art (http://servier.com/Powerpoint-image-bank). Servier Medical Art by Servier is licensed under aCreative Commons Attribution 3.0 Unported License.

Aging increases airway epithelium permeability, heightening susceptibility to environmental triggers and allergens, leading to inflammation and immune responses. AECs detect and respond to allergens by releasing cytokines that activate DCs. In the elderly, activated DCs produce proinflammatory cytokines, contributing to chronic airway inflammation. Dysregulated immune responses in aging airways result in a loss of immune tolerance, increasing susceptibility to lung tissue damage and hyper-responsiveness to antigens, potentially causing asthma or COPD (Figure 2(a)). Aging affects AEC and DC functions. In healthy-aged subjects, nasal epithelium exhibits reduced ciliary beat frequency and microtubular disarrangements^137^. Older adults have difficulty clearing teflon particles from small airways, taking over 21 days, leading to respiratory symptoms^138^. Age-related changes in the microbiome influence metabolite concentrations that affect immune cells, leading to increased inflammation. Understanding how these metabolites regulate DC function to maintain immune tolerance is crucial. In aged subjects, DCs often exhibit inflammatory responses to microbial metabolites^131^. Further research should investigate whether the DCs of aged subjects become immune to gut microbial metabolites or the microbiome, leading to a potential reversal of their immune system functions.

## Microbiome interventions in elderly care

Our recent study revealed that DCs from old mice (DC^old^) mice and DCs from dysbiotic mice (DC^dys^) exhibited diminished tolerance compared to DC from young mice (DC^young^). Specifically, DC^old^ and DC^dys^ displayed reduced capacity to induce Treg generation and regulate CD4 T cell activation. We observed a notable decrease in the prevalence of the *Lactobacillu*s genus in the gut of aged mice. To explore potential therapeutic approaches, we introduced *Lactobacillus plantarum* into the gut of elderly mice. This intervention successfully restored the tolerogenic function of DCs by modifying inflammatory and metabolic pathways. This novel finding sheds light on the impact of age-related gut dysbiosis on DC tolerance. Moreover, it offers promising insights into potential therapeutic strategies for addressing age-associated disorders through the use of *Lactobacillus plantarum*^7^.

A microbial mixture called IRT5, comprising *Bifidobacterium iridium, Streptococcus thermophilus, L. casei, L. reuteri*, and *L. acidophilus*, has shown promising results in experimental models of various diseases. In the context of rheumatoid arthritis, atopic dermatitis, and IBD, the administration of IRT5 increased the levels of IL-10, TGF-β, and IDO in DCs, leading to the induction of Tregs^139^. Additionally, IRT5 was found to be effective in reducing body trembling and weight loss in the myasthenia gravis model by blocking T cell-dependent B cell Ab responses against acetylcholine receptors (AchR)^140^. Furthermore, IRT5 supplementation resulted in decreased levels of AChR-specific IgG Abs, lymphocyte proliferation, and pro-inflammatory cytokines IL-17, IL-6, TNF-α and IFN-γ. DCs treated with IRT5 showed reduced production of pro-inflammatory cytokines in AChR-specific lymphocytes, accompanied by upregulation of arginase-1, RA-producing gene aldh1a2, IL-10 and TGF-β, indicating enhanced DC tolerance and induction of Tregs, which demonstrated protective effects^60,140^. Probiotics are currently under investigation for various diseases and have shown promising outcomes in preclinical and clinical studies. Diets rich in *Lactobacillus* have been associated with a reduced incidence of colon cancer^141^. Moreover, our laboratory’s research demonstrated that replenishing *Lactobacillus plantarum* restored tolerogenic behavior in DCs of aged and dysbiotic mice^7^. Probiotics are also gaining interest for their potential role in altering tumor apoptosis and proliferation, offering promising alternatives to radiotherapy and chemotherapy^142^.

## Conclusion

DCs play a critical role in maintaining self-antigen tolerance, and their dysfunction significantly impacts the health of elderly individuals. With advancing age, the gut microbiome undergoes changes that profoundly affect the immune system, particularly impairing DC function. This age-mediated dysbiosis creates an inflamed microenvironment, increasing the risk of autoimmune and inflammatory responses. The immune system undergoes a series of changes during aging, shifting the balance toward innate immune responses rather than adaptive ones. Intestinal DCs are particularly affected by these aging-related alterations. Cross-talk between the gut microbiome and intestinal DCs is crucial in maintaining tolerogenic behavior. Studies are exploring probiotic preparations and immune checkpoint inhibitors for cancer management, holding promise for future therapeutic approaches. Further research is needed to address better age-related disorders like COPD, cystic fibrosis, lung infections, Alzheimer’s disease, and Parkinson’s disease.

Although we have highlighted the impact of aging and gut dysbiosis on the immune system, especially focusing on DCs and the gut-lung axis, much remains to be explored. Unraveling the underlying mechanisms responsible for the loss of DC tolerance, both intrinsic and extrinsic, warrants investigation in preclinical and clinical models. Probiotics have emerged as significant players and have demonstrated promising results in preclinical and clinical studies. Future research should concentrate on developing optimal compositions of beneficial microbes that could aid elderly subjects in managing pathophysiological conditions. Strategies for monitoring gut health could prove valuable in detecting and addressing health alterations in elderly individuals at an early stage. Given the vulnerability of older subjects to infections and chronic diseases like age-related cognitive impairments, addressing their healthcare needs becomes a crucial aspect in managing their quality of life.

## Future prospects of early diagnosis of diseases and delaying aging by exploiting gut microbiota

Aging exerts a profound influence on the gut microbiota composition, consequently impacting immune system function. To comprehensively understand age-related changes in the gut microbiome, systematic studies are imperative. Unraveling the complicated relationship between specific gut microbes and their association with disease susceptibility or prevention could provide valuable insights for remedial interventions. Of particular interest is exploring the correlation between specific gut microbes and their role in disease susceptibility.

**Figure 3.**
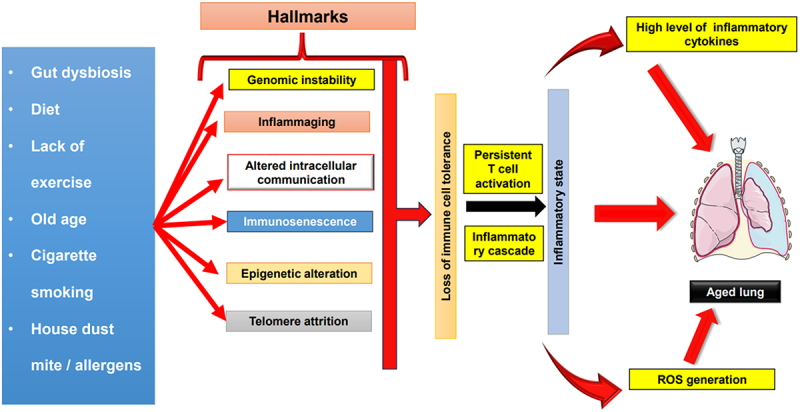
Role of inflammation and other hallmarks in driving lung aging. Numerous factors, including gut dysbiosis, aging, cigarette smoking, exposure to allergens, dietary patterns, and sedentary lifestyles, collectively contribute to a spectrum of immunological phenomena. These encompass immunosenescence, genomic instability, inflammaging, alterations in intracellular communication, epigenetic modifications, and telomere attrition. This intricate web of influences invariably results in the erosion of immune cell tolerance. The culmination of these hallmarks further translates into the loss of tolerance among DCs subsequently giving rise to sustained T cell activation. Consequently, triggers an augmented production of pro-inflammatory cytokines and ROS, ultimately fostering an inflammatory state. The consequences of this chronic inflammation, in the context of the respiratory system, precipitate the phenomenon known as lung aging. The figure is party adapted^143^.The artwork used in this figure was adapted from Servier Medical Art (http://servier.com/Powerpoint-image-bank). Servier Medical Art by Servier is licensed under aCreative Commons Attribution 3.0 Unported Licen.

Numerous distinct hallmarks, encompassing immunosenescence, genomic instability, inflammaging, altered intracellular communication, epigenetic modifications, and telomere attrition, collectively contribute to the deterioration of immune cell tolerance^143^. This complex cascade culminates in the establishment of a chronic inflammatory state, which plays a pivotal role in the process of lung aging (Figure 3). While the phenomenon of lung aging is multifaceted and characterized by various contributing factors, the underlying mechanism remains enigmatic. Future investigations are imperative to elucidate the precise roles played by DCs and other immune cells in mediating this inflammatory state and their involvement in the overall process of lung aging.

The presence or absence of certain bacteria may serve as indicative markers of disease protection or vulnerability, enabling early disease diagnosis through gut microbiome analysis. However, the cultivation of non-culturable bacteria remains a significant challenge, underscoring the need for research to identify the metabolites secreted by these microbes for potential disease treatment.

Moreover, comparative analysis of the gut microbiota in young and older adults could unveil microbes responsible for maintaining youthfulness. This intriguing concept presents the possibility of using these microbes or their products as therapeutics to delay or reverse aging and promote youthfulness, though unconventional, holding promise for future research and therapeutic interventions. Such investigations have the potential to revolutionize the field of aging and open new avenues for age-related disease management.
